# Metastatic Dissemination: Role of Tumor-Derived Extracellular Vesicles and Their Use as Clinical Biomarkers

**DOI:** 10.3390/ijms24119590

**Published:** 2023-05-31

**Authors:** Ilaria Giusti, Giuseppina Poppa, Giulia Di Fazio, Sandra D’Ascenzo, Vincenza Dolo

**Affiliations:** Department of Life, Health and Environmental Sciences, University of L’Aquila, Via Vetoio–Coppito 2, 67100 L’Aquila, Italy; ilaria.giusti@univaq.it (I.G.); giuseppina.poppa@graduate.univaq.it (G.P.); giulia.difazio1@student.univaq.it (G.D.F.); sandra.dascenzo@univaq.it (S.D.)

**Keywords:** extracellular vesicles (EVs), cancer, metastasis, pre-metastatic niche, liquid biopsy, cancer biomarkers

## Abstract

Cancer is a major cause of mortality in humans; often, rather than the primary tumor, it is the presence of metastases that are the cause of death. Extracellular vesicles (EVs) are small structures released by both normal and cancer cells; regarding the latter, they have been demonstrated to modulate almost all cancer-related processes, such as invasion, angiogenesis induction, drug resistance, and immune evasion. In the last years, it has become clear how EVs are widely involved in metastatic dissemination as well as in pre-metastatic niche (PMN) formation. Indeed, in order to achieve a successful metastatic process, i.e., penetration by cancer cells into distant tissues, the shaping of a favorable environment into those distant tissue, i.e., PMN formation, is mandatory. This process consists of an alteration that takes place in a distant organ and paves the way for the engraftment and growth of circulating tumor cells derived from the tumor primary site. This review focuses on the role of EVs in pre-metastatic niche formation and metastatic dissemination, also reporting the last studies suggesting the EVs role as biomarkers of metastatic diseases, possibly in a liquid biopsy approach.

## 1. Introduction

Cancer cells of solid tumors can remain where the tumor starts, i.e., at the primary site, or move towards different tissues, i.e., originate metastasis in a secondary site. Metastatic spreading can occur by several mechanisms: most commonly, the tumor cells reach the metastatic site by entering the bloodstream or lymphatic system, while less frequently, metastasis forms in the nearby tissues.

Metastases occurrence deeply affect the patient’s outcome, making it the main discriminating factor between low-risk and high-risk cancers: the first ones can be treated by surgical removal or active surveillance, while the second ones require stronger treatment [1].

Metastasis formation relies on several overlapping processes played out by tumor cells that also involve a complex interaction with host factors. Even though some of these steps are challenging and very inefficient, metastases still occur and remain the principal cause of cancer lethality.

In order to metastasize, one or more cancer cells need to acquire the ability to overcome some chemical and physical obstacles that would remain insuperable for other non-metastasizing tumor cells: cancer cells have to detach from the primary site and migrate through the extracellular matrix (ECM), remodeling it, by switching from an epithelial to mesenchymal cell type and move towards the secondary site where they have to proliferate and colonize, meanwhile resisting the host immune response and physical barriers. When metastasis by blood stream intravasation occurs, travel through the blood circulation, survival of shear stress, adhesion to the endothelium of the secondary site, and extravasation are also involved [1,2,3].

All these steps require the ability of tumor cells to communicate with other tumor cells or host cells by soluble or EV-associated factors.

Extracellular vesicles (EVs) are small membrane-enclosed structures that are released by all cells, including cancer cells; their cargo, associated or not with the plasma membrane, consists of many lipids, nucleic acids, and proteins that reflect the composition of the parental cell [4]. They are widely involved in many physiological and pathological processes as they represent an important means of communication between different cells; in particular, in tumors, EVs are able to support many of the processes that sustain tumor growth (proliferation, migration, evasion from apoptosis and immune surveillance, and drug resistance) [5].

Plenty of evidence, to date, suggest the contribution of EVs to the conditioning of premetastatic niches (among others by increasing vascular permeability, or priming the resident cells of secondary sites, or creating a hospitable niche); this review aims primarily to provide an overview of the metastatic process and EVs, and their general role in tumor progression. Subsequently, the role of EVs in the various phases of the metastatic process will be specifically discussed. Finally, current knowledge on the role of EVs as biomarkers of metastatic dissemination will be illustrated, also mentioning other therapeutic potential linked to their use.

## 2. Metastatic Processes

Cancer is a major cause of mortality in humans and, often, it is the presence of metastases that causes death, rather than the primary tumor. This is rather surprising, considering that metastatic dissemination is a highly inefficient process, as only a very small percentage of cells detaching from the primary tumor actually manage to cross endothelial barriers, bear the blood shear stress, evade the immune surveillance, and, finally, establish in a distant tissue to generate metastases. It has become clear, over the last few years that, for metastasis to successfully occur, cancer cells must find a favorable microenvironment at the site where they settle; it is also clear that tumor cells themselves can modify the microenvironment in distant organs to make it favorable to their successful settling [6]. This distant, favorable microenvironment is called the “pre-metastatic niche” (PMN). PMN should be considered as a cancer-favorable microenvironment not still invaded by tumor cells; in contrast, the metastatic niche is the microenvironment modulated by resident tumor cells that came from the primary tumor [7].

It is widely recognized that pre-metastatic and metastatic niche formation results from combined stimulation by both soluble factors and extracellular-vesicle (EVs)-associated molecules released by tumor cells [8,9].

The preparation of PMN relies on multiple processes, such as:

- Platelet activation: increasing evidence supports the fact that tumor cells can interact with platelets and that tumor-activated platelets play several roles in cancer metastatic dissemination, including the formation of an early metastatic niche by preparing a fertile soil for cancer cell mestastisis [10,11,12,13,14]. In a mouse model of lung cancer, the platelets-derived chemokines CXCL-5 and CXCL-7 attract granulocytes, contributing to the creation of an early metastatic niche; indeed, the inhibition of these chemokine receptors prevented granulocyte recruitment, thus impairing metastasis formation [15]. The role of platelets as mediators of communication between tumor cells and bone, before metastasis formation, has also been suggested; prostate cancer and melanoma cells are able to stimulate bone formation in distant sites (the stimulation of the bone turnover could benefit the metastasis, as cancer cells have been demonstrated to be more prone to bone colonization during the bone remodeling process) and platelet depletion inhibited this process, suggesting a key role of platelets for contributing to the generation of a tumor-favorable microenvironment [16]. Similarly, platelet involvement in PMN formation was also described for bone metastasis colonization by breast cancer cells, although it was based on different molecular mechanisms: the secretion of autotaxin by activated platelets, and its subsequent binding to tumor cell integrin ανβ3, promoted the formation of lysophosphatidic acid, which induced the osteoclast-mediated bone destruction, thus controlling the early stages of bone colonization [17]. The role of tumor-derived EVs (tEVs) in stimulating platelet activation is starting to be understood; interestingly, once activated, platelets, in turn, can release platelet EVs (pEVs), whose role in tumor biology is becoming clear, including their involvement in metastasis formation [18].
- Vascular leakiness: normal endothelial cells provide a physical barrier controlling the transfer of fluids, proteins, and cells from the blood to tissues and vice versa. Normally, endothelial cells are tightly connected by adherent and tight junctions, while tumor-associated vessels are featured by increased vessel hyperpermeability supported by endothelial fenestrae, transcellular holes, loosened inter-endothelial junctions, and an irregular basement membrane [19,20,21]. This impaired vascular barrier function and the associated vascular leakage are considered to be crucial for controlling the movement of cancer cells from primary sites to the blood (intravasation) and from blood to metastatic sites (extravasation) [7,22] (see below). Many soluble or EV-associated molecules and many signaling pathways are involved in the induction of a defective endothelium, such as miRNAs, VEGF, SDF-1, and angiopoietin-like molecules [23,24,25].
- Anti-tumor immunity: NK and T cells can attack transformed cells acting as a natural shield against cancer; tumor cells, indeed, are “altered self” cells that express “non-self” antigens triggering immune cells, thus being threatened by immune defense [26]. To break through this protective barrier, tumor cells play out several mechanisms (some of them based on EVs) at metastatic sites; first, being able to evade the immune system and, second, educating immune cells to establish a pro-inflammatory microenvironment that supports tumor growth and metastasis [9,27,28,29].
- Education of neighboring cells: it is known that once the tumor has formed, it starts to modify the surrounding stroma to become tumor supporting; even cells in distant organs are a common target of this education activity, including CAFs, endothelial cells, or TAMs; this “priming” process, triggering the formation of PMN, can be sustained by soluble mediators released by tumor cells, as well as by tumor-derived EVs, as they are able to travel through the blood and other biological fluids [9,28,30,31,32]. It has also been shown that EVs contribute to the organ-specific tropism of metastases by integrins [33,34,35].

Overall, PMN provides a favorable microenvironment for tumor cells, which are then able to promote metastatic niche formation. One of the main changes concerns the extracellular matrix (ECM). Indeed, even if its remodeling begins during the formation of PMN, the greatest changes occur in the metastatic niche and, among its various mechanisms, it has been shown that collagen hydroxylation enhanced by the hypoxic transcriptional factor HIF-1α enables metastatic growth [36].

Another crucial consideration concerns metabolism: in fact, depending on the “colonized” organ, the available nutrients vary and lead to organotropism of particular categories of cancer cells towards specific organs [32,37].

Once the environment in PMN is favorable to cancer cells, they start migrating consistently through the circulation, reaching the target tissue. To ensure that this process is as efficient as possible, cancer cells undergo several steps:

- Epithelial-to-Mesenchymal Transitions (EMT): a variety of transcription factors lead cancer cells to lose their epithelial phenotype and consequently their polarity, as well as the expression of adhesion molecules; this allows cells to move individually and acquire a fibroblast-like phenotype. The main characteristic of this transition is the increased level of invasiveness, motility, and ECM-degradation ability that the cells acquire [38].
- Invasion and intravasation: to establish a successful metastatic process, cancer cells need to invade neighboring tissues by breaking the basement membrane and infiltrating themselves. A variety of ECM components such as collagen, fibronectin, and glycoprotein are involved in the invasion of tumor cells, which can migrate and invade as groups of cells, or singularly, towards blood vessels, through the primary site tissue [3]. After invasion, tumor cells undergo an intravasation process, which is driven by the establishment of an intra-tumor hypoxic environment and involves the enrolment of cells, such as CAFs and TAMs, that assist ECM modification to foster the intravasation of tumor cells in blood circulation. Intravasation is supported by invadopodia development in cancer cells. Once entered into blood circulation, cancer cells are also known as circulating tumor cells (CTCs) [39,40,41].
- Resistance to death: once CTCs enter the bloodstream, they may be detected and attacked by NK cells; however, this process is inhibited by platelets, which coat circulating CTCs and protect them from being attacked through platelet factors that are released. Furthermore, CTCs are also resistant to the anoikis pathway due to an alteration in the expression of integrins and in metabolism. CTCs could also die as a result of the fluidic forces generated in the circulation; to avoid this, CTCs can form clusters that generate resistance to damage [3,13,42,43].
- Extravasation: for CTCs to leave circulation and reach the target organ, it is necessary for the cells to pass through the endothelium in the target tissue. This passage is ensured by platelets, cytokines, and other factors released by cells, which cause weakening of the endothelial barrier. In this way, through the formation of new invadopodia, CTCs can leave the circulation and settle on their target [36,44,45,46].

All of the processes, from the preparation of PMN to the settlement of metastasis, as explained above, rely on factors secreted by tumor cells, which include both soluble factors and EV-associated factors.

## 3. Extracellular Vesicles

Extracellular vesicles (EVs) represent a heterogeneous group of spherical particles enclosed in a phospholipid bilayer released by all cells and detected in all biological fluids, such as blood, urine, saliva, and cerebral and synovial fluids [47].

Depending on their biogenesis and size, EVs are commonly classified into three main groups: exosomes (EXOs), microvesicles (MVs), and apoptotic bodies (ABs). EXOs have dimensions between 50–150 nm and originate from the endo−lysosomal pathways: the process begins with the inward budding of the plasma membrane, leading to the formation of early endosomes. Their maturation generates late endosomes, whose further membrane invagination leads to the formation of intraluminal vesicles (ILVs) and, consequently, the formation of the so-called multivesicular bodies (MVBs). In the final step, MVBs fuse with the plasma membrane, releasing ILVs in the extracellular space; once released outside the cells in the extracellular microenvironment, ILVs constitute EXOs.

On the other hand, MVs have dimensions from 50 nm to 1000 nm and originate from direct outward budding of the plasma membrane [48,49].

The synthesis of EXOs and MVs is driven by different molecular mechanisms for the two groups; for EXOs, biogenesis can be carried out by two distinct pathways through the ESCRT system: the ESCRT-dependent pathway involves its subunit (ESCRT0, ESCRT I, ESCRTII, and ESCRT III) in membrane remodeling and leads to the formation of ILVs. The ESCRT-independent pathway entails the depletion of ESCRT machinery and the hydrolysis of sphingomyelin into ceramide, which in turn is involved in membrane shaping. Unlike EXOs, not much is known about the molecular pathways driving the biogenesis of MVs. However, the process can be distinguished into several steps that include membrane phospholipid rearrangement, external translocation of phosphatidylserine residues, and contraction of the cytoskeleton protein system [48,50,51,52].

ABs are the largest EVs (up to 5000 nm) and originate from cells having undergone apoptosis. Thus, they are the final consequences of the lysis of apoptotic cells and are formed by organized and sequential processes such as the plasma membrane blebbing followed by the formation of membrane protrusions and their cleavage is caspase mediated to release ABs. They contain organelles and chromatin, along with proteins and lipids [53]. As EXOs, MVs, and ABs can partially overlap in terms of size, their dimension cannot be used alone to determine their cell origin; moreover, specific markers for EVs subtypes are lacking. Thus, when it is not possible to determine their biogenesis, EVs can be classified based on their physical characteristic as “small EVs” (sEVs) and “medium/large EVs” (m/lEVs) when their size is, for example, lower or higher than 100–200 nm, respectively [47].

Although the discovery of EVs can be traced back to coagulation studies of the 1940s–1960s, during which they were described as “platelet dust”, only more recently have their roles in cell biology been established [54,55,56]. Nowadays, from the literature, it is possible to ascertain how EVs are involved in different processes that regulate cellular homeostasis, although how these occur still needs to be fully clarified. What is well known is the key role that EVs have in intercellular communication, as they are released by donor cells and absorbed by recipient cells, where EVs release their cargo, which includes proteins, lipids, DNA, and different types of coding and non-coding RNAs (mRNA, miRNA, circRNA, and lncRNA) [57,58,59].

Several physiological functions have been attributed to EVs. For example, it has been shown that the EVs secreted by oligodendrocytes can regulate the biogenesis of the myelin membrane [60], or provide metabolic support to neurons and neuroprotection [61]. In the the epidermis, EXOs derived from keratinocytes contain miRNAs that are involved in the processes of melanogenesis, and consequently in the regulation of skin pigmentation [58,62]. EVs can also be involved in immunity regulation and contribute to immune response, enhancing or suppressing it [63,64]. Indeed, in innate immunity, EVs derived from macrophages, NK cells, and neutrophils can mediate host recognition and its elimination. In adaptive immunity, on the other hand, EVs can activate B cells for antibody responses and provide direct and indirect antigen-specific stimulation to T cells [65]. EVs can also be caught up in waste management; taking over waste committed for disposal, they are thrown out from the cell, and then recognized by phagocytes, which discard them from circulation [66].

In addition to physiological processes, EVs are also involved in various pathologies that can affect different organs [67,68,69], and, above all, they play a crucial role in cancer. Bearing in mind that the content of EVs reflects that of the cell from which they originate, tEVs have a large impact on cancer hallmarks [70,71], such as sustaining proliferative signaling [72,73], resisting cell death [74,75], inducing angiogenesis [76,77,78], and evading the immune response [79,80]. In particular, most of the studies in the literature highlight the impact that EVs have in the modulation of the tumor microenvironment (TME), by generating an environment that promotes and supports the tumor, as, during the formation of the primary tumor, tumor cells can communicate both with each other and with neighboring cells, sending them soluble or EVs-associated signals as cytokines, signaling proteins, and other molecules. In this context, many studies have shown how tEVs can functionally modify bystander or distant fibroblasts, giving them a pro-tumorigenic phenotype known as CAFs [81,82,83]. As has already been said, in addition to promoting tumor formation and growth, tEVs ensure that TME also supports the settlementation of cancer cells in sites different from the primary tumor, facilitating the formation of a premetastatic niche, sustained by increased inflammation and vascular permeability, decreased activity by immune cells, and activation of the stromal cells [84].

EVs released by tumor cells have been proposed to support tumor invasiveness and encourage metastasis formation by preparing a pre-metastatic niche [85,86,87]. tEVs seem to express unique combinations of integrins that determine organotropic metastasis; EVs, especially EXOs, have been proven to be up-taken by organ-specific cells, thus “fertilizing” the soil to establish the pre-metastatic niche [33,34,35].

## 4. Role of Extracellular Vesicles in Pre-Metastatic Niche Formation and Metastatic Dissemination

**Figure 1:**
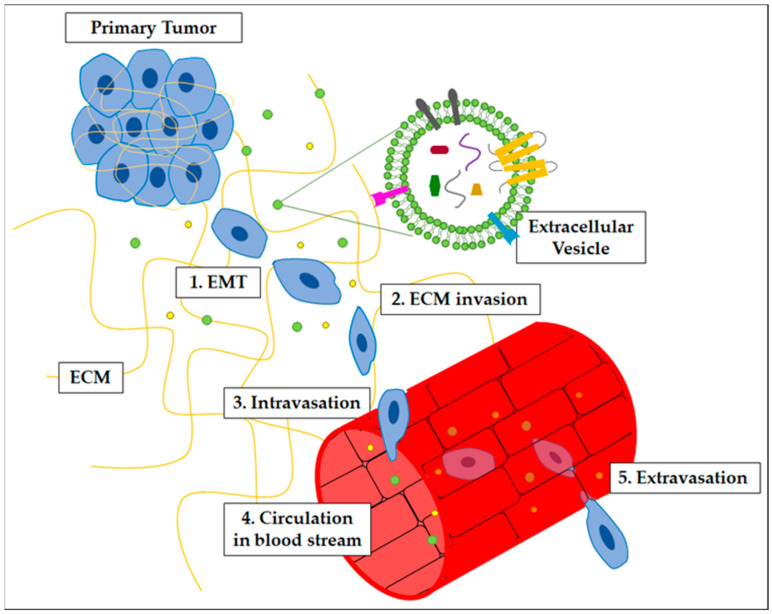
Schematic view of EV participation in the metastatic process, involving several steps: (1) epithelial-to-mesenchymal transitions (EMT); (2) ECM invasion; (3) intravasation; (4) circulation in the blood stream; (5) extravasation.

EVs contribute to many of the processes required for pre-metastatic niche formation and metastatic dissemination (Figure 1), as listed above.

*Epithelial-to-Mesenchymal Transition.* EVs can take part in EMT and invasion [88]. Some authors have identified several proteins (such as casein kinase II α and annexin A2) linked to EMT in the EVs released from the bladder carcinoma cell line [89]; melanoma cell-derived exosomes promoted an EMT-resembling process via MAPK (mitogen-activated protein kinase), but miRNAs miR-191 and let-7a were also found to be involved in this process [90].

*Resistance to death.* Once cells detach from ECM via EMT, there is the risk of triggering the anoikis process, against which cancer cells can acquire resistance. In this context, it was observed that EVs can be involved in this resistance: mir-210, which is elevated and associated with metastasis recurrence in colorectal cancer, was significantly up-regulated in exosomes released by colon cancer cells and is correlated to anoikis resistance [91]; similarly, EXOs derived from the A549 gemcitabine-resistant non-small-cell lung cancer cell line carry miR-222-3p, which enhanced the anti-anoikis features of parental gemcitabine-nonresistant cells by targeting the promoter of SOCS3 [92].

*Invasion.* It has been found that tumor cells release exosome-associated Hsp90α, which is involved in plasmin activation; these EXOs increase the plasmin-mediated motility of cells, thus contributing to cell invasion [93]; EXOs from MDA-MB-231 tumor breast cancer cells, which carry miR-10b, induce invasion in non-malignant HMLE mammary epithelial cells [94]; EXOs from tumor breast cancer cells with different metastatic potential (MCF-7, MCF7 Rab27b-transfected, and MDA-MB-231) administered to target cells (the same cells from which the EXOs were isolated or others), highlighting an increase in cell motility measured using a wound healing assay [95]; CD63- and Rab27a-positive EXOs released by tumor cells at invadopodia also facilitate cell invasion, with their secretion being critical for invadopodia formation and function; the authors suggested that the process was likely dependent on proteinases associated to EXOs [96]; A549 cells administered with lung adenocarcinoma EXOs-associated miR-1260b showed increased invasion [97].

*Intravasation and extravasation: impairing of the endothelium barrier.* EVs can be incorporated by endothelial cells, thus impairing endothelial functions by weakening the endothelium’s ability to maintain its barrier function. To reach distant sites, EVs alter vascular permeability, easing tumor cell entry into the tissues [9,98,99].

Many studies have focused on the role of miRNAs as being responsible for inducing endothelial barrier leakiness, by targeting molecules in endothelial cells that are involved in the inter-cellular adhesions: for example, miR-939 loaded in breast cancer EXOs targets and down-regulates VE-cadherin, thus loosening the endothelial barrier and favoring, in vitro, the trans-endothelial migration of cancer cells [100]; in a similar in vitro model, miR-105 targets ZO-1 (a tight junction protein), impairing the endothelial cell barrier, as demonstrated by in vitro permeability assays and trans-endothelial electrical resistance (TEER) measure [101]; ZO-1 is also targeted by miR-23a, released by hypoxic lung cancer cells through EXOs [102]; miR-181c transported by EVs released from brain metastatic breast cancer cells downregulates PDPK1 and induces an anomalous actin organization and an altered localization of actin, N-cadherin, and tight junctions proteins (Claudin-5, Occludin, ZO-1) in endothelial cells, thus causing the breakdown of the blood−brain barrier (BBB), the barrier that is composed of specialized endothelial cells and separates the blood from the brain tissue [103]. Hepatocellular carcinoma EV-associated miR-103 similarly affected endothelial cell permeability and facilitated the trans-endothelial invasion of tumor cells in vitro, but also promoted metastasis formation in vivo; in this case, miRNA targeted molecules involved in endothelial junctions proteins, such as VE-cadherin, ZO-1, and p120 catenin [104].

VEGF-A loaded in EVs, released by ex vivo cultured patient-derived glioblastoma stem-like cells, can also play a role in inducing higher permeability of the endothelium, as demonstrated in a model of glioblastoma multiforme [105]; in a similar model, it is the Semaphorin3A expressed on the surface of EVs isolated from patient-derived glioblastoma cells that induces an increased vascular permeability [106]

*Platelet activation.* The role of platelet activation in the preparation of metastatic niche raised the question of whether tEVs could be involved in this process too.

Several studies have shown that tumor-derived MVs and EXOs can be enriched with tissue factor (TF), a protein primarily involved in blood coagulation initiation. In an in vitro model of breast cancer cells, the EVs were more enriched in TF in the metastatic cell line MDA-MB-231 than in the non-metastatic MCF7 cell line and contributed to accelerating coagulation. Interestingly, when TF was shuttled from MDA-MB-231 to MCF7 cells by EVs, this resulted in a significant increase in TF activity in MCF7 cells [107]; EVs can also activate platelets in a TF-independent way [108].

The platelet-activation role of tumor EVs can rely on further pathways. One of them involves the formation of NETs, web-like structures composed of DNA and proteins that are involved in platelet activation: tEVs isolated from the murine 4T1 mammary carcinoma cell line induced NETs formation in G-CSF-primed neutrophils, supporting the idea that tumor cells cooperate with neutrophils through EVs, thus favoring a thrombotic state favorable to cancer progression and metastasis [109].

*Alteration of immune response.* As already said, tumor cells take advantage of EVs to overcome anti-tumor immunity in the pre-metastatic niche.

EVs derived from melanoma cells, for example in vitro, compromise the correct maturation of dendritic cells (DCs) by negatively regulating monocyte maturation (the DCs, in the lymph nodes, are crucial to detect and present tumor-associated antigens to lymphocytes); moreover, EVs treatment of DCs downregulates some chemokines (FLT3L, IL-15, MIP-1α, and MIP-1β) compared with the control, suggesting an alteration of DC functions mediated by melanoma EVs [110].

The ability of EXOs to alter immune microenvironments at distant organs was also demonstrated in vivo in a breast cancer mouse model. EXOs released by metastatic (EO771 and 4T1) and not-metastatic (67NR) murine breast cancer cells, when intravenously administrated to mice, was predominantly distributed to the lung, which is a frequent site of metastasis for breast cancer, and were internalized by CD45^+^ bone-marrow-derived immune cells. EXOs from the metastatic cell line EO771 altered the immune asset by decreasing the frequency of CD8 T cells and NK cells, altering the relative composition of CD4 cells and increasing the gMDSC population (MDSC cells have already been shown to create a permissive PMN, generating an immunosuppressive microenvironment). The immunosuppressive effect of EXOs was further confirmed; in fact, once conditioned for 30 days with EXOs from the metastatic cell lines, mice administrated with tumor cells developed a higher metastatic burden in the lung compared with the control mice. The same authors showed that breast-cancer-derived EXOs directly impacted T-cell and NK-cell functions by suppressing the proliferation of CD8 and CD4 T cells and reducing the cytotoxic activity of NK cells against target tumor cells [111].

Macrophage activity is likewise affected. In mouse models, studies of liver metastases have shown that tEVs carry LCFA; these EVs are then internalized by metastasis-associated macrophages, which undergo functional and metabolic alteration. Internalization is enhanced by the macrophages’ CD36 receptor, which affects fatty acid uptake and mediates lipid metabolism through immunosuppressive activity in TME by inhibiting CD8^+^ T cells [112]. The immunosuppressive activity of macrophages has also been evaluated in lung metastases; a study in mice models with subcutaneous tumors showed that the administration of tumor-derived EXOs led to an increase in PD-L1 and the inhibition of T-cell function, resulting in a reduced immune response. In addition to the change towards a pro-tumor phenotype, macrophages also incur a metabolic change, driven by NF-kB, which acts by increasing glycolysis, with a consequent increase in lactate production that enhances KRAS-driven tumor growth and contributes to the reduction in T-cells [113]. Immune suppression through PD-L1 has also been widely studied in breast cancer [114,115].

In addition to macrophages, neutrophils activity is modified in the metastatic process. In a lung cancer mouse model, it was shown that small nuclear RNAs loaded in tEVs upregulate the expression of TLR3 in the host lung epithelial cells; TLR3 expression and activation in these cells resulted in the recruitment of neutrophils that promote pre-metastatic niche formation: its deficiency, instead, reduced metastasis in the spontaneous cancer metastatic mouse model used. Interestingly, neutrophils seem to be involved in the suppression of innate and adaptive anti-tumor immunity by inducing the formation of an inflammatory pro-metastatic microenvironment [116].

*Dormancy*. A peculiar “trick” cancer cells can use to evade the immune system is to enter into a momentary dormant stage once PMN is reached [117,118]; indeed, in this regard, many studies have revealed that EVs influence the behavior of tumor cells at sites distant from the primary tumor, mainly through the transfer of miRNAs. For example, bidirectional communication between breast cancer cells and bone marrow MSCs fosters metastasis after a quiescent state. The latter is driven by the release from MSCs of EXOs containing miR-222 and miR-223, which are responsible for the survival of some tumor cells that reach a dormant state and are, thus resistant to pharmacological treatments [119]. In addition to the miRNAs cargo of EVs just mentioned, miR-127 and miR-197 have also been identified as also being responsible for cycle arrest into the G0-phase of breast cancer cells [120].

In chronic myelogenous leukemia, through EVs trafficking, bone-marrow cells release miRNA-126 and are involved in the dormant stage process of leukemia stem cells [121].

*Stroma and stromal cells modulation in the pre-metastatic niche.* Similar to what happens in the primary site of a tumor, cancer-released EVs can also contribute to stroma alteration in the pre-metastatic niche.

EXOs derived from malignant pancreatic ductal adenocarcinoma (PDAC) lesions contribute to liver pre-metastatic niche formation; “exosome education” (exosomes administered daily for 3 weeks to mice) enhanced metastatic burden in the liver. On the contrary, the “exosome education” performed with EXOs from normal cells did not induce this process, strongly suggesting a specific role for tumor EXOs in the generation of PMN. In the liver, PDAC EXOs are taken up by Kupffer cells, but not by fibroblasts, or epithelial or endothelial cells; Kupffer cells educated with EXOs were induced to produce TGF-β and other factors related to liver fibrosis that, in turn, induce the release of fibronectin by hepatic stellate cells. Then, macrophages and neutrophils are recruited into the liver by fibronectin deposits. It seems that the macrophage MIT is responsible for the observed results, as its knockdown prevents EXO-induced metastasis [31].

EVs-stimulated fibronectin secretion has been similarly described in other cancers [122]. The deposition of fibronectin by activated stromal fibroblasts in PMN has been highlighted as one of the key processes in pre-metastatic niche formation as its accumulation facilitates the recruitment of bone-marrow-derived cells, which are crucial components of the niche [7,9]. In primary sites of cancer, tumor cells contribute to stromal fibroblast activation into CAFs, by both soluble molecules and EVs-associated molecules (mainly TGF-β) [82,83,123]; it is reasonable to hypothesize that the action exerted by the EVs in this process may also have an effect on distant fibroblasts (i.e., in the sites of metastasis), given the ability of EVs to travel in biological fluids. Once activated, CAFs can, in turn, favor cancer cell progression [83,124,125,126].

In addition to being activated to modify the composition and architecture of the stroma in PMN, the fibroblasts are also educated by tumor cells to induce acidification of the niche; in fact, local acidification of the stroma is supposed to be related to PMN formation. Human adult dermal fibroblasts educated by melanoma-derived EXOs were metabolically reprogrammed to increase the aerobic glycolysis and decrease oxidative phosphorylation, thus acidifying the extracellular environment; miR-155 and miR-210 were proposed as being responsible for this observed effect [127].

Moreover, breast-cancer-derived EVs can interact with human primary mammary epithelial cells, generating a favorable microenvironment for incoming metastatic cells through several mechanisms (such as ROS increase induction, secretion of factors promoting breast cancer cell growth, and autophagy stimulation) [128,129]. Another interesting mechanism employed by breast cancer cells to favor permissive PMN formation is the suppression of glucose uptake in non-tumor cells, so that this nutrient becomes more available for tumor cells themselves; breast cancer cells, in fact, release high levels of EVs-enclosed miR-122 that suppresses glucose metabolism in receiving cells by downregulating pyruvate kinase and glucose transporter 1. This effect was observed in vitro in several recipient cells, typically abundant in the breast cancer premetastatic niche, such as lung fibroblasts, brain astrocytes, and neurons. Moreover, when intravenously administered to mice, breast cancer EVs were effectively taken up by brain and lung tissues, thus resulting in a reduced expression of pyruvate kinase and glucose transporter 1, and in the promotion of metastasis formation; the in vivo inhibition of miR-122 decreased the incidence of metastasis in the brain and lung by reactivating glucose uptake in distant organs [130].

In colorectal cancer (CRC) as well, fibroblasts undergo a change towards the pro-tumorigenic phenotype CAFs, supporting mainly lung and liver metastasis through the formation of PMN. The transition in CAFs is promoted by the transcriptional factor RUNX2, enhancing the release of tEVs containing, in their cargo, the integrin ITGBL1 which is responsible for epithelial−mesenchymal transition through the activation of fibroblasts in CAFs. Once activated, CAFs foster the NF-kB pathway, and through the secretion of cytokines, they promote tumor progression and the metastatic process [131].

Breast-tumor-cell-derived EXOs were also involved in the establishment of a pro-metastatic environment in the lungs; indeed, a study performed on a mouse model showed that breast-tumor-derived exosomes contain a high amount of miRNA, particularly miR-200b-3p, which is up taken by alveolar epithelial cells and triggers cellular microenvironment modification. In particular, miRNA inhibits PTEN, causing an increase in CCL2 chemokines responsible for pre-metastatic lung niche formation [132]. The increased expression of CCL2, as well as Ly6C + CCR2+ monocyte levels, is also enhanced in response to the neoadjuvant treatment of breast cancer with taxanes and anthracyclines: they lead to the secretion of tEVs enriched in Annexin-A6, which can activate NF-kB-dependent endothelial cells in an Annexin-A6-dependent manner. This mechanism results in the establishment of lung metastasis [133].

The ability of cancer-derived EXOs to educate the cells in PMN was also sustained by a study focused on the integrin repertoire expressed in exosomes. It was demonstrated that the integrin combination expressed on EXOs dictates the interaction with specific target cells in specific organs; for example, EXOs expressing ITGα_6_β_4_ and ITGα_6_β_1_ preferably bound to lung fibroblasts and epithelial cells, mediating the tropism to this organ. Moreover, EXO uptake induced the over-expression of inflammation-related genes of the S100A family in lung fibroblasts; as S100A4 is known to be a metastasis-promoting factor controlled by ITGα_6_β_4_ and to regulate lung metastasis, the authors concluded that this exosome-associated integrin is able to activate the Src–S100A4 axis in targeted lung fibroblasts during PMN formation, thus educating lung and preparing it for the growth of metastatic cells [33].

Furthermore, it has been shown that cholesterol homeostasis contributes to the signal transduction pathway of prostate-cancer-derived EVs in promoting PMN formation and metastatic dissemination in the bone marrow. It has been suggested that when a high amount of cholesterol is produced, tEVs are able to upregulate the NF-kB pathway, enhancing the differentiation of osteoclasts. This process allows the formation of PMN and for the promotion of the metastatic process in the bone [107].

In addition to the above-mentioned factors, EVs can be involved in the formation of PMN and metastatic dissemination by other mechanisms. For instance, it has been demonstrated that, in breast cancer, cell treatment with the chemotherapy drug Paclitaxel induces the release of tEVs that reach the lungs through the blood system; there, tEVs promote tissue permeability by modifying the ECM, and increasing several related proteins, mainly fibronectin. The modification of ECM decreases the rigidity of lung tissue, fostering an environment that supports cells in forming a PMN, easing the metastatic dissemination [134].

*Direct role in metastatic dissemination and metastasis formation.* Several studies conducted on in vitro or animal models support the direct role of EVs in metastatic dissemination.

In human pancreatic ductal adenocarcinoma tissues, the tumor tissues express lower levels of PRKD1 than non-tumor tissues; PRKD1 usually acts by inhibiting cell motility and its loss/reduction, which reduces the phosphorylation of cortactin (substrate for PRKD1), thus causing an increase in F-actin l at the plasma membrane, is associated with a strong increase in sEVs secretion. PRKD1 knocked-out mice are characterized by increased development of lung metastasis and, also, increased levels of sEVs release. The intravenous injection into Nod/Scid xenotransplant mice of these sEVs increased the development of metastasis to the lung; this observation is explained by EVs cargo, which is altered and carries the integrin α6β4, which specifically targets EVs to the lung, explaining the observed organ specificity [135].

In hepatocellular carcinoma (HCC), increased levels of miR-3129 carried by tEVs mediate EMT and metastasis formation. The mechanism behind this promotion is explained by the observation that miR-3129 can inhibit the activity of the tumor suppressor TXNIP. Indeed, its downregulation in HCC results in increased proliferation and decreased apoptosis, thus ensuring metastasis mainly to the lungs [136]. The treatment of mice with breast-cancer-cell-derived EXOs enriched in mir-105, which induces vascular permeability, significantly increased the distant metastasis development in the brain and lung, compared with treatment with EXOs derived from a “poor”-miR-105 cell line [101]. EXOs from gastric cancer cells enriched in CD97, usually overexpressed in gastric carcinomas, exhibited a metastasis-promoting capacity, as demonstrated both in vitro and in a footpad lymph node metastasis mouse model; on the contrary, EXOs lacking CD97 exhibited a lower ability to promote lymphatic metastasis, suggesting that CD97-loaded exosomes are key factors in metastasis and niche formation in gastric carcinoma [137].

The contribution of EVs to metastasis-related processes is summarized in Table 1.

## 5. Extracellular Vesicles as Biomarkers of Metastatic Diseases

As EVs are present in different body fluids and their cargo reflects that of the original cell, over time, precision medicine and personalized medicine have shown a growing interest in their potential as biomarkers for cancer diagnosis, prognosis, and for the development of targeted therapies. Although most biomarkers are related to monitoring tumor progression in its broadest sense, in the last few years, several works have shed light on molecules that could be considered potential biomarkers for patients with metastases (Table 2) [57,139,140,141].

A lot of studies suggest the use of EVs for cancer, specifically as metastasis biomarkers. Most of these studies are focused on EV-associated miRNAs or other proteins. In some cases, it is the very level of specific EVs populations that can function as a biomarker.

*miRNAs*. An examination of the peritoneal fluid in patients with gastric cancer revealed the presence of a higher level of some miRNAs, carried by EVs, related to the presence of peritoneal metastases, suggesting their potential use as biomarkers for the identification and development of treatments targeting these metastases [154].

In CRC, EVs are considered potential biomarkers for tumor progression, as well as for metastasis; EVs-associated mir-17a-5p is significantly up-regulated in CRC patients, especially in those with distant metastasis and higher clinical stages [144,146,155]. In the serum, higher levels of miR-934 carried by tumor-derived EXOs are also associated with CRC liver metastasis, suggesting their potential use as biomarkers; miR-934 can induce the polarization of M2 macrophages, downregulating the expression of PTEN and the activation of the PI3K/AKT pathway. Thus, it could promote metastatic niche formation and liver metastasis by the secretion of the chemokine CXCL13, activating the CXCL13/CXCR5/NFκB/p65 pathway and promoting an inflammatory microenvironment [145].

The analysis of the plasma levels of miR-21 contained in the cargo of sEVs has been correlated to liver metastases in patients with CRC; by binding to the TLR6 of macrophages, miR-21 can induce a pro-inflammatory condition of macrophages that secrete IL-6, thus supporting the onset of liver metastases. Therefore, this miRNA can be considered a potential biomarker for the prognosis and diagnosis of liver metastases related to CRC [146]. However, higher expression levels of miR-21 and others have also been found in serum EVs, defining their possible use as diagnostic biomarkers to distinguish metastatic from non-metastatic CRC [144,156]; for example, high levels of miR-181a-5p-enriched EVs were also found, and they are related to the establishment of liver metastases; the underlying mechanism appears to be induced by the persistent activation of hepatic stellate cells (HSCs) by miR-181a-5p, resulting in increased secretion of the chemokine CCL20, which in turn activates TME reprogramming and pre-metastatic niche formation in the liver [147].

miR-21 is also associated with metastasis in breast cancer: higher levels were found in patients who had bone metastases related to this type of cancer. It appears that miR-21 influences the differentiation and function of osteoclasts by regulating the expression of PDCD4; hence it could be useful as a biomarker for diagnosing breast-cancer-related bone metastases [148].

Stage II and III breast cancer patients exhibited blood EXOs enriched in miR-105 (known for its role in inducing vascular leakiness) and its levels were significantly higher in patients who later developed distant metastases, suggesting that miR-105, along with other blood markers (proteins or miRNAs), could likely allow for identifying breast cancer patients with a higher risk for metastasis [101]. Deregulation of other miRNAs present in plasma EVs was, instead, related to brain metastases associated with breast cancer in their early and late stages [150].

*EV-associated proteins.* Not only miRNAs, but also EV-associated proteins, could be considered as potential biomarkers; an example is given by a study conducted on non-small cell lung cancer (NSCLC), in which proteomic analysis highlighted how some liposaccharide-binding proteins contained in EXOs isolated from the serum differed between patients with metastases and patients without metastases, suggesting these proteins as prospective markers in the diagnosis of metastatic NSCLC [152].

Studies on mice and human blood samples have brought out a higher expression of oncoprotein MET in bone metastatic melanoma, already recognized for its roles in the various processes that lead to metastases. In this case, MET carried by tumor EXOs can influence and educate the bone marrow cells towards a pro-metastatic phenotype, suggesting its potential role as a biomarker of bone metastasis. The authors suggest that MET, along with other molecules (i.e., TYRP2, VLA-4, HSP70, and an HSP90 isoform) could represent a specific melanoma signature in circulating EXOs, in patients with advanced melanoma, that could be used as a metastatic disease indicator [10]. On the other hand, the plasma of pancreatic ductal adenocarcinoma patients (PDAC) contains EXOs expressing MIF in significantly higher levels in those patients whose disease progressed compared with healthy control subjects or patients with no evidence of disease 5 years after the first diagnosis. This suggest that exosome-associated MIF may be a marker for liver metastasis of PDAC [31]. Another potential PDAC-related biomarker of liver metastases is the CD44v6/C1QBP complex carried by HSC exosomes: higher levels in patients than in healthy people were found, and it appears to be involved in the activation of the IGF-1 signaling pathway, essential for PDAC progression, invasion, and chemoresistance, leading the authors to suggest that highly expressed EXOs associated with CD44v6 and C1QBP are promising biomarkers for predicting prognosis and liver metastasis in patients with this tumor [142].

In vivo and in vitro studies highlight how breast cancer EVs with a high content of CDH11 cadherin and ITGA5 integrin are responsible for setting up a pre-metastatic niche in bone. Niche establishment is supported by an increased expression of osteoblastic factor RUNX2, which educates osteoblasts to form an osteogenic pro-tumor environment. The authors suggest that, as the molecular features of EVs mirror the cell of origin, circulating tEVs associated with a high expression of CDH11high/ITGA5 could become promising diagnostic biomarkers able to predict bone metastasis using the liquid biopsy approach [149].

Finally, with the role of EXOs-associated integrins in determining organ tropism being demonstrated, it has been proposed that the integrins pattern of blood circulating tumour-derived EXOs may be used to predict the propensity to form metastases, but also which organ is most likely to be affected (suggesting their usefulness as “organotropism biomarker”); plasma evaluation of integrin exosome-associated ITGβ_4_ (already related to a propensity to determine lung metastasis) revealed increased levels of this integrin in EXOs from breast cancer patients with lung metastasis compared with patients with no metastasis or metastasis in another organ (liver). Similarly, the integrin ITGα_v_ (related to liver metastasis) was increased in EXOs isolated from the plasma of pancreatic cancer patients with liver metastasis, with respect to patients with no metastasis or lung metastasis; interestingly, upon diagnosis, the EXO-associated ITGα_v_ levels were higher in cancer patients who successively developed liver metastasis compared with control subjects or patients with no liver metastasis within three years of diagnosis [33,35].

Lungs are the most frequent sites of metastasis in salivary cystic adenoid carcinoma (SACC); it has been shown how integrin α2β1, transported by CAF-derived EVs, and detectable in the plasma at high levels, regulates the activity of lung fibroblasts towards a pro-tumorigenic condition, supporting the creation of a pulmonary pre-metastatic niche; thus, the authors suggest that EV-associated integrin β1 in the blood might be a promising biomarker to predict SACC metastasis [153].

*EVs levels.* In NSCLC, as well as in melanoma, the level of circulating EXOs positive for PD-L1 is higher in patients with metastasis compared with healthy controls, suggesting it may be a potential diagnostic marker [151,157,158]. PD-L1 is an immunosuppressive molecule that tumor cells exhibit on their surface that impairs the anti-tumor actions of T cells [151]. 

## 6. Discussion and Conclusions

It is evident, based on what has been discussed so far, that EVs can act as potential biomarkers for the diagnosis of tumor metastases. Surely, further studies are needed to identify target molecules transported by EVs that represent specific and unique markers for a given metastasis, and to consider EVs safe and effective tools in clinical applications.

It should be stressed, however, that this would only represent a further clinical application of EVs, as they have many therapeutic potentials. Actually, their ability to reach all body fluids, allowing for non-invasive or minimally invasive liquid biopsy (that allows for real-time monitoring), makes EVs ideal biomarkers for many clinical applications.

One of the most chased applications is their use as diagnostic biomarkers. Many research has outlined this approach: for example, the molecular composition of EVs has been shown to be useful for kidney disease diagnosis, and circulating miRNA levels reflect the decrease in eGFR (estimated glomerular filtration rate), suggesting their use as biomarkers of uraemia [159,160]; EV-associated α-synuclein is correlated with Parkinson’s disease [161]; some miRNAs are useful for the diagnosis of heart failure [162].

However, it should not be forgotten that EVs can also be used as a marker to evaluate the efficacy of a treatment and the related patient response: some EV-associated molecules, often miRNA, are correlated with a patient’s response to treatment [163].

In the oncology field, specifically, EVs have been widely studied as diagnostic biomarkers, even for early diagnosis, but also to evaluate the tumor status and the cell origin; many researchers have highlighted EXOs potentiality in glioblastoma or pancreatic cancer or lung cancer, to cite a few [164,165,166,167,168,169,170,171].

EVs can also be used as cell-free-therapy to induce tissue regeneration, as suggested by some studies [172]. Molecules transported by EVs have been demonstrated to be able to exert a neuroprotective effect (by suppressing apoptosis or enhancing myelin formation) [173] or they can protect against chronic kidney injury [174,175].

As EVs are naturally able to transfer their cargo to recipient cells, they hold great potential as drug delivery vehicles. The other available drug delivery systems, for example liposomes or micelles, lack some interesting features of EVs [176]: they induce immune rejection (EVs do not), have a poor targeting ability (contrary to EVs), and are characterized by low drug loading (that is why many methods are being developed to load useful materials into EVs [177]). Many studies have suggested the potential of EVs as a drug delivery system in tumor targeting [178].

Finally, some authors suggest that EVs can be used as vaccines, as they have a natural ability to act as antigen presenting [179]; from this point of view, EVs have some very appropriate features, such as a long circulating half-life and the natural ability to interact with specific target cells. Several approaches can be used for this purpose, for example, cells can be transfected to produce specific antigens in order to release EVs carrying those antigens in their native conformation, or EVs can be directly loaded with specific molecules [180]. Many papers describe these applications for EVs, which, lately, due to the pandemic, are focusing on COVID-19 [180,181,182,183]

Although it is clear from what has been said that the potential clinical applications of EVs are extremely fascinating and promising, it should be emphasized that there are still some crucial points to be resolved before their effective entry into the clinical routine [184].

Among the main issues to be resolved, there are certainly some technical problems concerning the isolation and storage of EVs. There are objective difficulties in isolating and purifying vesicles in clinical practice (there is no uniformity either in EVs isolation methods in research settings). Many techniques are available, each one with some limitations: ultracentrifugation is the most used and traditional method, but is time-consuming, generally has a low throughput, and requires complex equipment, and EVs can be contaminated by non-EVs components (proteins and lipoproteins); some more promising techniques include (i) precipitation by polymers addition: does not require complex equipment, but similarly to ultracentrifugation, cannot avoid the co-precipitation of contaminant proteins and sometimes requires very long time; (ii) size-exclusion chromatography allows for high EV purity, but EVs are highly diluted and this can be incompatible with the following analysis; (iii) magnetic capture using antibodies-coated magnetic beads is a very promising method (currently quite expensive) but only allows for the isolation of EVs that are positive for the antigen that is directed against the antibody [185].

Storage conditions of biofluids are another issue, as long-term storage requires freezing, which can affect the integrity of EVs [186].

Last, but not least, if we collect EVs samples (from blood or urine, for example) to be analyzed in a clinical laboratory, a whole series of issues relating to sampling and the pre-analytical phase must be added: in sample collection, timing must be carefully considered as the composition of EVs can be modified if the sample is kept in the test tube; before proceeding to the isolation of EVs, a pre-processing step to remove cells or cells debris will likely be necessary and it will be necessary to carefully evaluate which type of procedure to use for this purpose, in order to avoid the unwanted removal of some vesicles [185].

In conclusion, as we have delved deeper into the biogenesis and function of EVs, it has become increasingly clear that they have immense diagnostic, prognostic, and therapeutic potential in the context of cancer. However, these promises of EVs can only be fully realized by understanding their molecular cargo, as well as their heterogeneity, in order to allow for the establishment of therapeutic and diagnostic guidelines. Despite their potential, the difficulty in the large-scale production of purified EVs remains a challenge that must be overcome to increase their clinical application. In addition, even if EVs have gained a lot of attention in the field of drug delivery due to their biocompatibility and low immunogenicity, all these existing limitations of EV-based therapeutics must be addressed to establish EVs as viable therapeutic options. By utilizing optimal EV subpopulations for these applications, the promise of EVs in cancer therapy and diagnostics could be fully realized in the following years.

## Figures and Tables

**Table 1:** EVs and EV-associated molecules involved in metastasis-related processes.

EVs or EVs Associated Molecules	Cancer Type	Changes Detected	Refs.
casein kinase II α annexin A2	Bladder carcinoma	EMT induction	[89]
miR-191 let-7a	Melanoma	EMT induction	[90]
miR-210	Colorectal cancer	Resistance to death	[91]
miR-222-3p	NSCLC	Resistance to death	[92]
miR-10b	Breast cancer	Increased invasion	[94]
EVs	Breast cancer	Increased invasion	[95]
Proteinases	Breast cancer	Increased invasion	[96]
miR-1260b	Lung adenocarcinoma	Increased invasion	[97]
miR-939	Breast cancer	Down-regulation of VE-cadherin, increasing endothelial barrier permeability and cell migration	[100]
miR-105	Breast cancer	Dysfunction of the endothelial barrier due to ZO-1 alteration	[101]
miR-23a	Lung cancer	Dysfunction of the endothelial barrier due to ZO-1 alteration	[102]
miR-181c	Breast cancer	Downregulation of PDPK1 and ensuing the alteration of the BBB	[103]
miR-103	Hepatocellular carcinoma	Endothelial cell permeability and trans-endothelial invasion	[104]
VEGF-A	Glioblastoma	Endothelial permeability	[105]
Semaphorin3A	Glioblastoma	Vascular permeability	[106]
TF	Breast cancer	Accelerated coagulation platelet activation	[107,108]
tEVs	Mammary carcinoma	NETs formation in G-CSF-primed neutrophils, supporting a thrombotic state	[109]
EVs	Melanoma	Incorrect maturation of dendritic cells through the downregulation of chemokines	[110]
EXOs	Breast cancer	Immune system alteration by decreasing the CD8 T cells and NK cells frequency, altering the relative composition of CD4 cells, and increasing the gMDSC population	[111]
EXOs	Breast cancer	Alteration of T-cell and NK-cell functions by suppressing the proliferation of CD8 and CD4 T-cells and reducing the cytotoxic activity of NK cells against target tumor cells	[111]
LCFA	Liver metastasis	Immunosuppressive activity in the TME mediated by macrophage	[112]
EXOs	Subcutaneous tumors	Reduced immune response due to PD-L1 increasing and T-cell function inhibition	[113]
EVs	Breast cancer	Immune suppression driven by PD-L1	[114,115]
tEVs	Lung cancer	Upregulation of TLR3 in host lung epithelial cells, neutrophils recruitment, and formation of a pro-inflammatory state	[116]
tEVs	Breast cancer	Increasing tissue permeability by modifying the ECM, through increasing in several proteins, such as fibronectin	[134]
tEVs	Prostate cancer	Upregulation of the NF-kB pathway, enhancing the differentiation of osteoclasts in bone in response to the high amount of cholesterol	[138]
miR-222 miR-223	Breast cancer	Establishment of a dormant state and resistance to pharmacological treatments	[119]
miR-127 miR-197	Breast cancer	Cycle arrest into the G0-phase	[120]
miR-126	Chronic myelogenous leukemia	Dormant stage of leukemia stem cells	[121]
EXOs	Pancreatic ductal adenocarcinoma	Mestastatic burden in the liver induced by the production of TGF-β and other factors and the recruitment of macrophages and neutrophils	[31]
TGF-β	Primary tumors	Stromal fibroblast activation into CAFs	[82,83,123]
miR-155 miR-210	Melanoma	Acidification of the extracellular environment due to the increase in aerobic glycolysis and decrease in oxidative phosphorylation in human adult dermal fibroblasts	[127]
miR-122	Breast cancer	Suppression of glucose metabolism in receiving cells through the downregulation of pyruvate kinase and the GLUT1	[130]
ITGBL1	Colorectal cancer	EMT caused by fibroblasts activation in CAFs	[131]
miR-200b-3p	Breast cancer	Cellular microenvironment modification by the inhibition of PTEN, causing an increase in CCL2 chemokines	[132]
Annexin-A6	Breast cancer	Increased expression of CCL2, and Ly6C + CCR2+ monocytes levels	[133]
ITGα6β4 ITGα6β1	Lung cancer	Activation of the Src–S100A4 axis in targeted lung fibroblasts	[33]
ITGα6β4	Pancreatic ductal adenocarcinoma	Increase in sEVs secretion related to reduced PRKD1 concentration	[135]
miR-3129	Hepatocellular carcinoma	Inhibition of the tumor suppressor TXNIP activity	[136]
mir-105	Breast Cancer	Vascular permeability	[101]
CD97	Gastric cancer	Cell migration	[137]

**Table 2:** Potential biomarkers carried by EVs for metastasis.

Primary Cancer and Related Metastasis	Biomarker	Refs.
Liver metastasis in pancreatic cancer	ITGαv	[33]
Lung metastasis in breast cancer	ITGβ4	[35]
Breast Cancer metastasis	miR-105	[101]
Liver metastasis in pancreatic ductal adenocarcinoma	MIF CD44v6/C1QBP	[31,142]
Peritoneal metastasis in gastric cancer	miR-21-5p miR-92a-3p miR-342-3p miR-223-3p	[143]
Colorectal cancer metastasis	miR-17a-5p miR-17-92 miR-934	[144,145]
Liver metastasis in colorectal cancer	miR-21 miR-181a-5p	[146,147]
Breast Cancer bone metastasis	miR-21 CDH11 ITGA5	[148,149]
Breast Cancer brain metastasis	miR-92a-1-5p miR-205-5p miR-181a-1-3p miR-802-5p miR-194-5p	[150]
Non-small cell lung cancer metastasis	Liposaccharide-binding proteins PD-L1	[151,152]
Melanoma metastasis	PD-L1	[151]
Bone metastasis in melanoma	MET	[10]
Lung metastasis in salivary cystic adenoid carcinoma	α2β1	[153]

## Data Availability

Not applicable.

## Abbreviations

C1QBP: Complement component 1 Q subcomponent-Binding Protein; CAF: Cancer-Associated Fibroblast; CCL: Chemokine (C-C motif) Ligand; CDH11: Cadherin-11; CXCL: Chemokine (C-X-C motif) Ligand; CXCR: Chemokine (C-X-C motif) Receptor; FLT3L: FMS-Like Tyrosine Kinase 3 Ligand; G-CSF: Granulocyte Colony Stimulating Factor; gMDSC: Granulocytic- Mieloid-Derived Suppressor Cells; HIF-1α: Hypoxia-Inducible Factor-1α; HSP70: Heat Shock Protein 70; HSP90: Heat Shock Protein 90; IGF-1: Insulin-like Growth Factor 1; IL: Interleukin; ITG: Integrin; ITGBL1: Integrin Subunit Beta Like 1; KRAS: Kristen Rat Sarcoma Virus; LCFA: Long-Chain Fatty Acid; MIF: Macrophage migration Inhibitory Factor; MIP: Macrophage Inflammatory Protein; MIT: Migration Inhibitory Factor; NET: Neutrophil Extracellular Traps; NF-kB: Nuclear Factor kappa-light-chain-enhancer of activated B cells; NK: Natural Killer; PDCD4: Programmed Cell Death Factor 4; PD-L1: Programmed Death- Ligand 1; PDPK1: Phosphoinositide-Dependent Kinase-1; PI3K/AKT: Phosphatidilinositol-3 Kinase/AKT; PRKD1: Protein Kinase D1; PTEN: Phosphatase and Tensin homolog; ROS: Reactive Oxygen Species; RUNX2: Runt-Related Transcription Factor 2; SDF-1: Stromal Cell- Derived Factor 1; TAM: Tumor Associated Macrophage; TLR: Toll-Like Receptor; TXNIP: Thioredoxin-Interacting Protein; TYRP2: Tyrosinase-Related Protein 2; VE-Cadherin: Vascular Endothelial- Cadherin; VEGF: Vascular Endothelial Growth Factor; VLA-4: Very Late Antigen-4; ZO-1: Zonula Occludens-1.
